# Emerging arboviruses and public health challenges in Brazil

**DOI:** 10.1590/S1518-8787.2016050006791

**Published:** 2016-06-17

**Authors:** Tamara Nunes Lima-Camara

**Affiliations:** Departamento de Epidemiologia. Faculdade de Saúde Pública. Universidade de São Paulo. São Paulo, SP, Brasil

**Keywords:** Arbovirus Infections, epidemiology, Communicable Diseases, Emerging, prevention & control, Insect Vectors, Public Health

## Abstract

Environmental modification by anthropogenic actions, disordered urban growth, globalization of international exchange and climate change are some factors that help the emergence and dissemination of human infectious diseases transmitted by vectors. This review discusses the recent entry of three arboviruses in Brazil: Chikungunya, West Nile, and Zika virus, focusing on the challenges for the Country’s public health. The Brazilian population is exposed to infections caused by these three arboviruses widely distributed on the national territory and associated with humans. Without effective vaccine and specific treatment, the maintainance and integration of a continuos entomological and epidemiological surveillance are important so we can set methods to control and prevent these arboviruses in the Country.

## RESUMO

A modificação do ambiente por ações antrópicas, o crescimento urbano desordenado, o processo de globalização do intercâmbio internacional e as mudanças climáticas são alguns fatores que vêm facilitando a emergência e disseminação de doenças infecciosas humanas transmitidas por vetores. Este comentário aborda a recente entrada de três arbovírus no Brasil, Chikungunya (CHIKV), West Nile (WNV) e Zika (ZIKV), com enfoque nos desafios para a Saúde Pública do País. Transmitidos por mosquitos vetores amplamente distribuídos no território nacional e associados ao homem, a população brasileira encontra-se exposta à infecção por esses três arbovírus. Na ausência de vacina eficaz e tratamento específico, são importantes a manutenção e integração de uma vigilância entomológica e epidemiológica contínua, a fim de direcionarmos métodos de controle e prevenção contra essas arboviroses no País.

## INTRODUCTION

Infectious diseases have some peculiarities that distinguish them from other human diseases, such as the unpredictable and explosive character on a global level, transmissibility, close relationship with both the environment and human behavior, and the ability to be prevented and eradicated^10^. Most of the pathogens responsible for human infectious diseases have zoonotic origin, i.e., they are kept in nature in cycles involving one vector and one wild animal (e.g., monkey or bird). However, due to anthropogenic actions associated mainly with economic activities, many insect vectors, such as mosquitoes, have become synanthropic, favoring the transmission of pathogens to humans^20^. Thus, over the last 10 years, we have seen the emergence of some diseases transmitted by mosquitoes, especially arboviruses such as Chikungunya, West Nile, and Zika virus, in different countries of the Americas (Figure A and B).

**Figure:**
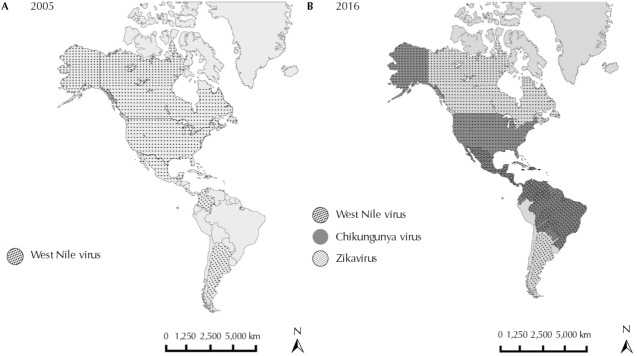
Emerging Arboviruses in the Americas. Distribution of West Nile (dotted), Chikungunya (dark gray), and Zika virus (hatched) in the Americas: (A) in 2005, and (B), in 2016.

Sources: Centers for Disease Control and Prevention – CDC, Chikungunya Virus Home. Geographic distribution. Atlanta: CDC; 2016 [cited 2016 Jan]. Available from: http://www.cdc.gov/chikungunya/geo/index.html; Centers for Disease Control and Prevention – CDC, Chikungunya Virus Home, Areas with Zika. All countries and territories with active Zika virus transmission. Atlanta: CDC; 2016 [cited 2016 Jan]. Available from: http://www.cdc.gov/zika/geo/active-countries.html

In addition to the interference and modification of ecosystems by human action, other factors are related to the emergence of arboviruses in those countries, such as disordered urban growth and processes of globalization and expansion of international exchange, as well as climatic changes^16^. The population moves voluntarily for work, study or leisure, or involuntarily, as refugees, after a natural disaster or during a war in their country. These population movements increase the risk of travellers to carry pathogens not yet detected in other areas, or even new serotypes or strains more resistant to a particular virus already known, causing the emergence or reemergence of a disease^1^.

Global warming is also an important factor in the transmission dynamics of pathogens to humans. Global temperature increase affects the mosquito vectors by reducing the development time of the larvae and, thus, increasing rapidly the population of adults. It also decreases the extrinsic incubation period, i.e., the time for the virus to reach the mosquito’s salivary gland, making it suitable to transmit this etiologic agent^11^. Additionally, studies indicate that global warming may expand the distribution of diseases involving vectors, both in altitude and latitude^9^. Although tropical countries present positive social, environmental, and climatic conditions for transmission of new infectious diseases, the circulation of some arboviruses is also being observed in some temperate climate countries.

The aim of this comment was to discuss the recent entry of the arboviruses Chikungunya (CHIKV), West Nile (WNV), and Zikavirus (ZIKV) in Brazil, focusing on the challenges for the Country’s public health.

### Arboviruses and their Vectors

#### Chikungunya virus

Chikungunya fever is caused by a virus that belongs to the *Alphavirus* genus of the *Togaviridae* family. CHIKV was first isolated in 1952/1953, during an epidemic in East Africa (Tanzania and Mozambique). The term “Chikungunya” is derived from the Makonde language, spoken in some areas of northern Mozambique and southern Tanzania, which means “that which bends up”, referring to the posture gained by the patient due to severe arthralgias^14^. Other than strong arthralgia, symptoms such as high fever, headaches, nausea and vomiting can also occur^14^.

This arbovirus did not receive much attention until 2005, when outbreaks struck some Indian Ocean islands, as the French island Reunion, where more than 240,000 people were infected and 203 died^2^ ^,^ ^14^. In 2006, Chikungunya outbreaks occurred in India and in some Southeast Asian countries, while in 2007, autochthonous cases were reported in Ravenna, Italy, as well as in the south of France, which also reported the disease in 2010^14^.

In Africa, the cycle of CHIKV occurs mainly in forested areas, involving wild mosquitoes of the *Aedes* genus, such as the *Aedes furcifer-taylori*, e.g., and nonhuman primates^17^. In Asia, the transmission cycle of CHIKV was usually associated with the urban mosquito *Aedes (Stegomyia) aegypti* and with humans. However, during the epidemic of Chikungunya in the Reunion island, low numbers of *Ae. aegypti* and high density of *Aedes (Stegomyia) albopictus* were found in the area^2^. The analysis of the genetic material sequence of CHIKV showed a specific mutation in the envelope protein of this virus (E1-A226V). This point mutation of CHIKV increased its ability to infect *Ae. albopictus*, turning this mosquito into an excellent vector for humans in several areas where *Ae. aegypti* is absent^14^ ^,^ ^28^.

At the end of 2013, the first case of local transmission of CHIKV was reported in the Americas, in the Caribbean region. Just a year later, at the end of 2014, South American countries, such as French Guiana, Venezuela, Colombia, Suriname, Paraguay, and Brazil had already reported the local circulation of CHIKV (CDC, 2014) ^a^. The first report of local transmission in Brazil occurred in 2014 in the city of Oiapoque, Amapa, and currently this state has one of the highest case numbers in the Country, as the same occurs in Bahia and Pernambuco. Although the main vector of CHIKV in Brazil is still unknown, a recent study proved that both Brazilian populations of *Ae. aegypti* and *Ae. albopictus* show high vector competence, which makes this arbovirus a potential threat to the Country^29^.

#### West Nile virus

West Nile fever is caused by a virus that belongs to the *Flavivirus* genus of the *Flaviviridae* family. WNV was first isolated in a feverish woman in the West Nile district of Uganda, Africa, in 1937. Then, some outbreaks of this arbovirus were reported elsewhere in Africa, Middle East, Asia, and Europe^25^.

The entry of WNV in the West occurred in late August and early September 1999, in New York City, United States of America. The city had a human encephalitis outbreak, which indicated the presence of an arbovirus of the *Flavivirus* genus as etiologic agent^18^. Associated to human encephalitis, in the same area, viral encephalitis of unknown etiology reached resident birds, especially crows. Initially, this encephalitis was diagnosed as St. Louis (SLEV), but the viral sequencing, isolated from the brain of a sick bird, showed that it was WNV^19^. Between 1999 and 2005, about 19,525 cases of West Nile fever was reported in the United States, 8,606 being neuroinvasive cases, with 771 deaths^13^. In 2009, 720 cases were reported, with 32 deaths^17^. WNV spread across the United States so quickly that the country was considered endemic for West Nile virus in only 10 years^18^.

Most patients infected with WNV are asymptomatic, but the symptoms include fever, headache, nausea, and vomiting. Generally, the recovery is complete, but weakness and fatigue may last longer. Less than 1.0% of infected people develop the most aggressive form of the disease, showing severe neurological problems, such as meningitis and encephalitis, which may lead to death. Such manifestations are more common in older adults and immunosuppressed people^19^ ^,^ ^27^.

The WNV cycle is maintained in nature by birds and mosquitoes, while humans and horses are considered final hosts, as they do not act as infectious sources for the vector^18^. Although WNV has been isolated in some species of *Aedes* and *Anopheles* in Africa, Europe, and United States, undoubtedly, the *Culex* genus encompasses the main mosquito vectors of this arbovirus, including *Cx. quinquefasciatus* ^18^.

In South America, serological evidence of WNV was detected in horses and birds in Colombia, Venezuela, and Argentina. In Brazil, the first serological evidence of WNV activity occurred in 2009, in the Pantanal region, state of Mato Grosso do Sul, with the virus isolation in horses^25^. Recent studies confirm the circulation of this arbovirus in equines, especially horses, in that same region^23^ ^,^ ^26^. At the end of 2014, the first human case of West Nile virus was reported in the state of Piaui.

#### Zika virus

Also belonging to the *Flavivirus* genus of the *Flaviviridae* family, ZIKV was first isolated in rhesus monkeys, in 1947, in the Zika forest, Uganda. The isolation of ZIKV in humans was confirmed in Nigeria, but some serological evidence of human infection by this arbovirus have also been reported in other African countries, such as Egypt, Tanzania, Gabon, and Sierra Leone, as well as in Asian countries, such as India, Malaysia, Thailand, and Indonesia^12^. In 2007, a Zika virus outbreak hit the Yap island, Micronesia, in the Pacific Ocean, whereas French Polynesia, also in Oceania, reported a major epidemic of the disease in October 2013. Thus, the circulation and transmission of ZIKV out of the African and Asian continents were confirmed^12^.

In 2015, Brazil reported the first autochthonous human cases of Zika, confirming the recent entry of this arbovirus in the Country. Bahia and Rio Grande do Norte were the first Brazilian states to report cases of ZIKV infection^3^ ^,^ ^4^ ^,^ ^32^. However, in technical note, Rio de Janeiro’s State Government also confirmed cases of this arbovirus in the cities of Sumare, state of Sao Paulo, and Rio de Janeiro, state of Rio de Janeiro, until May 31, 2015^b^. Currently, the autochthonous transmission of ZIKV occurs in 21 states: Alagoas, Amazonas, Bahia, Ceara, Federal District, Espirito Santo, Goias, Maranhao, Mato Grosso, Mato Grosso do Sul, Para, Paraiba, Parana, Pernambuco, Piaui, Rio Grande do Norte, Rio de Janeiro, Rondonia, Roraima, Sao Paulo, and Tocantins^c^.

The most common form of ZIKV transmission is by the bite of infected female mosquitoes of the *Aedes* genus, *Ae. aegypti* being the main vector in Brazil. The symptoms of this arbovirus appear a few days after the mosquito bite, they last from three to 12 days and include low-grade fever, arthralgia, myalgia, headache, conjunctivitis, and maculopapular rash^3^ ^,^ ^7^ ^,^ ^32^.

## Public health challenges in Brazil

The emergence of arboviruses in places that were safe represents a potential challenge for public health in many ways.The recent entry of CHIKV, WNV, and ZIKV in Brazil and in other countries of the Americas exposes the population to the infection, once all individuals are susceptible; in addition, no vaccines are available as prophylactic method and no antiviral drugs are effective for the treatment^5^. The entry of these arboviruses in dengue-endemic countries, such as Brazil, may collapse the health services during simultaneous explosive epidemics. Furthermore, the economic impact of these new arboviruses is alarming because, although most patients infected with CHIKV and WNV present full recovery after the acute phase of the disease, some symptoms may last for weeks or months, such as strong arthralgia of Chikungunya and extreme fatigue of West Nile virus, interfering in the occupational activities of the patient^14^ ^,^ ^27^. On the other hand, ZIKV infection may develop a syndrome of autoimmune origin and neurological order in the patient, called Guillain-Barré, causing generalized muscle weakness and paralysis^21^. Additionally, we suspect that ZIKV infection in pregnant women can be associated with the recent outbreak of microcephaly in Brazilian newborn babies, which increases the urgent need to implement a health surveillance related to this infection^22^.

The noteworthy association of mosquito vectors with humans is also a challenge. *Ae.aegypti* and *Cx. quinquefasciatus* have wide distribution in Brazil and are extremely associated with humans and the urban environment^6^. In addition, *Ae. albopictus* is present in almost all national territory^24^, it can be found inrural and suburban environments, using artificial or natural containers to reproduce. Therefore, the contact of humans with these vectors is common and frequent across the Country, increasing the risk for epidemics in different states. The efficient control of these mosquitoes has been challenging. Regarding *Ae.aegypti*, for instance, new technologies have been used to control this dengue vector, Chikungunya, and Zika in Brazil, such as the release of genetically modified adults or mosquitoes infected by the *Wolbachia* bacteria. However, more research is required to confirm the effectiveness of these methods^31^.

The environment also represents an obstacle to the control of these vectors. Disordered urban growth, combined with the pollution of rivers and ditches, provides artificial oviposition sites for the proliferation and dissemination of mosquitoes, especially of *Ae. aegypti* and *Cx. quinquefasciatus*. Climate change also contributes to the proliferation of mosquito vectors. The increased rainfall frequency, which has been observed in some places, entails the accumulation of water in more containers, increasing the supply of natural or artificial breeding sites for female mosquitoes to deposit their eggs. On the other hand, the period of drought in certain areas forces people to store water in barrels or other artificial deposits, which serve as breeding sites for the proliferation and increase of vector populations^17^.

Other relevant challenge for public health is the diagnosis of these new arboviruses. In Brazil, several arboviruses circulate, such as Mayaro (MAYV), Venezuelan Equine Encephalitis (VEEV), Eastern Equine Encephalitis (EEEV), Rocio (ROCV), and Dengue virus (DENV), which present many symptoms similar to those observed for CHIKV, WNV, and ZIKV^15^. In addition, some serologic tests used for the detection of these arboviruses in vertebrate hosts can present cross-reaction, complicating the accurate diagnosis^25^. Recently, MAYV was identified in patients during dengue outbreaks in Mato Grosso^30^. Thus, the diagnosis based on clinical-epidemiological examination, or even by serological analysis, can be difficult.

Finally, despite the lethality of CHIKV and ZIKV being considered low, cases of coinfection with other arboviruses have been reported in patients from other continents^8^, which makes us more attentive to patients’ diagnosis, as well as to the study on the interaction of these viruses in humans.

The recent entry of new arboviruses challenges physicians, health professionals, and researchers to perform an active and ongoing investigation about specific symptoms and serology of vectors, etiological agents, and of environmental and social factors that may be associated with the epidemics and the emergence of new cases. Thus, the strengthening and integration of entomological and epidemiological surveillances is necessary, so we can set methods to control and prevent these diseases in the Country.

## Footnotes

a Centers for Disease Control and Prevention – CDC, Chikungunya nowcast for the Americas. Nowcast: Chikungunya in the Americas. Atlanta: CDC; 2015 [cited 2016 Apr 5]. Available from: http://www.cdc.gov/chikungunya/modeling/index.html

b Rio de Janeiro, Secretaria de Estado da Saúde, Subsecretaria de Vigilância em Saúde. Nota Técnica sobre Vigilância do Zika Vírus. *Nota Tecnica SVS*. mar. 2015. Available from: http://www.riocomsaude.rj.gov.br/Publico/MostrarArquivo.aspx?C=WfmHPQJ5e3w%3d

c Portal da Saúde – SUS. Novos casos suspeitos de microcefalia são divulgados pelo Ministério da Saúde. *Boletim*. 14 jan. 2016. Available from: http://portalsaude.saude.gov.br/index.php/cidadao/principal/agencia-saude/21677-novos-casos-suspeitos-de-microcefalia-sao-divulgados-pelo-ministerio-da-saude
